# Burosumab in infants with X-linked hypophosphatemic rickets: a case series

**DOI:** 10.1186/s13023-025-04177-2

**Published:** 2026-01-08

**Authors:** Ravit Regev, Avivit Brener, Nitzan Dror, Raphael Krespi, Rebeca Rapalino, Efrat Chorna, Ophir Borger, Adar Lopez, Yael Lebenthal, Leonid Zeitlin

**Affiliations:** 1 The Institute of Pediatric Endocrinology, Diabetes and Metabolism, Dana-Dwek Children’s Hospital, Tel Aviv Sourasky Medical Center, Tel Aviv, 6423906 Israel; 2 The School of Medicine, Faculty of Medical & Health Sciences, Tel Aviv University, Tel Aviv, Israel; 3 Pediatric Endocrinology Unit, Meir Medical Center, Kfar-Saba, Israel; 4 Bone Disease Unit, Pediatric Orthopedic Department, Dana-Dwek Children’s Hospital, Tel Aviv Sourasky Medical Center, Tel Aviv, Israel; 5 Center for Oral Health, Tel Aviv Sourasky Medical Center, Tel Aviv, Israel; 6 Social Services, Tel Aviv Sourasky Medical Center, Tel Aviv, Israel; 7 The Nutrition & Dietetics Unit, Tel Aviv Sourasky Medical Center, Tel Aviv, Israel

**Keywords:** X-linked hypophosphatemic rickets, XLH, Burosumab, Infants, Linear growth, Rickets score, Phosphate levels, Skeletal health

## Abstract

**Background:**

X-linked hypophosphatemic rickets (XLH) is a rare inherited metabolic bone disorder caused by excess fibroblast growth factor 23 (FGF23), leading to hypophosphatemia and rickets. Burosumab, a human monoclonal antibody targeting FGF23, was approved for the treatment of XLH in April 2018. By 2022, the FDA extended its approval to include children as young as six months of age.

**Objectives:**

To describe three infants with XLH who began burosumab therapy before one year of age and were monitored for at least one year.

**Design:**

Case series.

**Methods:**

Clinical outcomes, including anthropometric measures, skeletal outcomes (Rickets Severity Score [RSS], mechanical axis deviation [MAD], and neck-shaft angle [NSA]), and laboratory parameters, were assessed in a real-world setting.

**Results:**

Two patients demonstrated satisfactory linear growth, and one experienced growth faltering, possibly due to sleep-disordered breathing or phosphate imbalance. These patients received higher doses of burosumab than the current guideline recommendations for achieving treatment goals aimed at normalizing phosphate levels. The patient’s phosphate levels improved but did not normalize. Bone pain was not formally assessed, but parents reported improvements in their children’s conditions. Importantly, two patients with assessable mechanical axes demonstrated neutral mechanical axis deviations, indicating improved lower limb alignment and supporting the therapeutic efficacy of burosumab. All three patients had favorable RSS outcomes, and none developed long bone diaphyseal bowing or coxa vara following this early initiation of burosumab treatment.

**Conclusion:**

This case series demonstrated potential benefits of early initiation of burosumab treatment for XLH by showing improvements in growth, phosphate levels, and skeletal outcomes. Burosumab appears well tolerated in infancy, but further research is needed to refine dosing strategies and assess its long-term safety and therapeutic efficacy in young patients with XLH. Meticulous monitoring and individualized care are essential throughout treatment.

**Plain language summary:**

This case series followed three infants with X-linked hypophosphatemic rickets (XLH) who initiated burosumab treatment at 6 months of age, filling a significant knowledge gap in early intervention. Despite requiring higher-than-recommended dosing, burosumab demonstrated favorable outcomes in terms of growth parameters and skeletal development over a minimum one-year follow-up. Two patients maintained normal growth trajectories, whereas one experienced temporary growth faltering, which resolved after adenoidectomy. Importantly, neutral mechanical axis deviation was achieved, indicating effective prevention of lower limb deformities. No significant adverse effects were observed. Despite limitations such as a small sample size and short follow-up, these findings suggest that early burosumab therapy can improve outcomes in infantile XLH patients. Further research is needed to refine dosing protocols and assess long-term safety and efficacy in this young population.

## Introduction

X-linked hypophosphatemia (XLH) is a rare inherited progressive metabolic bone disorder caused by mutations in the phosphate-regulating endopeptidase homolog on the X chromosome (*PHEX*) gene, leading to elevated fibroblast growth factor 23 (FGF23) levels, renal phosphate wasting and impaired bone mineralization [1, 2]. Children with XLH typically present with rickets skeletal deformities, disproportionate short stature, bone pain, and dental complications [1].

Conventional treatment with oral phosphate and active vitamin D has been used for decades but is limited by incomplete efficacy, side effects, and frequent dosing requirements [1, 3, 4]. Burosumab, an anti-FGF23 monoclonal antibody, offers a targeted treatment and has demonstrated superior outcomes compared to conventional therapy in children over one year of age [4–6]. In 2022, burosumab received FDA approval for use in children as young as six months; however, data on its effectiveness and safety when started in infancy remain scarce [7].

This study aims to evaluate the effects of initiating burosumab therapy before 12 months of age on linear growth, rickets severity, and biochemical markers in infants with XLH. The report explores whether early treatment may contribute to improved growth trajectories and skeletal outcomes in a real-world clinical setting.

## Patients and methods

Three infants with XLH followed at the Metabolic Bone Clinic at Dana-Dwek Children’s Hospital, Tel Aviv Sourasky Medical Center, and the Endocrine Unit at Meir Medical Center began burosumab treatment before the age of one year. During the first six months of therapy, they were evaluated every 2–4 weeks to allow close monitoring of clinical status, laboratory parameters (including serum phosphate and alkaline phosphatase), and potential adverse effects. Once clinical stability was achieved, follow-up visits were scheduled every three to six months. At each visit, medical interviews were performed to assess treatment adherence and clinical response, and to monitor for adverse effects. Skeletal radiographs were obtained every 6 to 12 months as part of routine clinical care to evaluate rickets severity. Treatment decisions were guided by a composite assessment of clinical findings, biochemical markers, and radiographic outcomes.

The three patients were started on conventional therapy with oral phosphate and active vitamin D shortly after diagnosis, in accordance with standard XLH treatment guidelines [5]. Conventional therapy was discontinued seven days before the initiation of burosumab, which was administered at a dose of 0.8 mg/kg of body weight, rounded to the nearest 10 mg according to the protocol [8]. The dosage was gradually adjusted on the basis of laboratory results, in accordance with practice guidelines [9]. Clinical characteristics, biochemical markers, findings on skeletal radiographs, and growth parameters were collected and analyzed.

Weight was measured with a tabletop electronic infant scale with an attached measuring rod (Model 374 + Measuring Rod 233, SECA, Gmbh & Co., Germany), and crown-heel length was measured with the infant in the supine position. The World Health Organization (WHO) Growth Standards were used for infants 0 to two years of age, and z-scores were tallied [10]. The weight-to-length ratio was calculated as an indicator of weight status in children younger than two years of age [11]. Body mass index (BMI) of older children was calculated by dividing body weight in kilograms by height in meters squared, and height, weight, and BMI z-scores were determined using sex- and age-specific CDC 2000 growth charts [12].

Laboratory blood samples were collected prior to burosumab injections and analyzed for serum levels of phosphorus, calcium, creatinine, alkaline phosphatase (ALP), 25-hydroxy vitamin D, 1,25-dihydroxyvitamin D (1,25 vit D), intact parathyroid hormone (PTH), and intact FGF23. Spot urine tests were performed to measure phosphorus, calcium, and creatinine levels. Total reabsorption of phosphate (TRP) was calculated by mean of an online calculator and is presented as a percentage [13]. The tubular maximum transport of the phosphate-to-glomerular filtration rate (TmP/GFR) was calculated from values in serum and spot urine as previously described elsewhere [14]. Skeletal radiographs were obtained as needed for routine clinical care and evaluated by two independent bone health specialists (RR and LZ) who used the Thacher Rickets Severity Score (RSS) [15]. The images were scored on a scale from 0 to 6, with higher scores indicating greater severity of rickets. The mechanical axis of the lower limb, also referred to as the Mikulicz line, was determined by drawing a line from the center of the femoral head to the center of the ankle. Under normal conditions, this axis passes approximately 8 mm medial to the midpoint of the knee. Deviations from this range signify alignment abnormalities. Specifically, an axis that shifts laterally indicates valgus alignment, whereas a medial shift suggests varus alignment. The extent of this deviation is termed the mechanical axis deviation (MAD) [16]. The femoral neck angle, a radiological measurement that refers to the angle formed between the axis of the femoral neck and the axis of the femoral shaft [17], was assessed by a pediatric orthopedist (R.K.).

### Case series

Demographic, genetic, and clinical characteristics of all three patients are summarized in Table 1

**Table 1:** Sociodemographic, perinatal, genetic, and clinical characteristics of three infants with XLH

	Patient 1 5 y 2 mo ♀	Patient 2 4 y 3 mo ♀	Patient 3 1 yr 10 mo ♀
**Demographic**			
Ethnicity	Sephardic/Ashkenazi Jewish	Ashkenazi Jewish	Ashkenazi Jewish
Family member with XLH	None	Mother	None
**Perinatal history**			
Pregnancy	In vitro fertilization, dizygotic twins	Spontaneous	Spontaneous
Maternal medical history	None	XLH	GDM, Factor XI deficiency, Lynch syndrome, CMV-positive
Mode of delivery	Cesarean section, elective	Cesarean section due to prolonged labor	Cesarean section, elective
Gestational age, weeks	35 + 5	38	38 + 4
Birth weight, grams	2686	2630	4015
Birth weight, %, z score	59 (0.22)	21 (−0.81)	98 (2.02)
Perinatal course	Uneventful	Respiratory distress hospitalization in NICU; normal phosphate levels	Born LGA no hypoglycemic episodes
**Genetic analysis**			
Age at genetic diagnosis	19 days; biochemical confirmation CMA in amniocentesis *PHEX* mutation in 1 twin	3 months; maternal XLH and patient’s low phosphate level	Prenatal; whole exome sequencing
Genetic alteration	Not available	Genetic testing not done	De novo chrx:22208575 c0.1601c > t p.pro534leu rs886041363
**Clinical history**			
Congenital malformation	None	None	Ventricular septal defect
Developmental milestones	Normal	Developmental delay	Normal
Other medical conditions	Severe allergic reaction; unknown allergen, epi-pen	Growth faltering; adenotonsillectomy. mild hearing loss	Mild hearing loss

**Patient 1.** This 5-year 2-month-old female presented to our clinic shortly after birth. She was born preterm at 35 + 5 weeks of gestation, with a birth weight appropriate for gestational age (2.685 kg), as one of a pair of twins. Prenatal genetic testing via amniocentesis identified a *PHEX* mutation, although it was initially unclear which twin was affected. At 19 days of age, the patient was hospitalized for neonatal fever. Blood tests revealed hypophosphatemia, and further evaluation demonstrated elevated FGF23 levels, confirming the diagnosis of XLH. Anthropometric measurements and laboratory evaluation at the time of diagnosis are presented in Table 2. Head circumference at birth was not available, and length was not measured at that time.

**Table 2:** Growth, metabolic, and skeletal characteristics at the initiation of therapy

	Patient 1 5 y 2 mo ♀	Petient 2 4 y 3 mo ♀	Patient 3 1 yr 10 mo ♀
**Conventional therapy**			
Age at initiation of conventional treatment	2.1 months	3.5 months	2 days
Weight, kg %, z score	5.130 92 (1.42)	4.820 7 (−1.46)	3.730 91 (1.31)
Length, cm %, z score	54.6 56 (0.16)	57.8 14 (−1.07)	not done
Wt-to-length, %, z score	92 (1.41)	15 (−1.03)	
**Serum metabolic markers**			
iFGF23, pg/mL (range 28–37)	< 21	87	Not done
Calcium, mg/dL (range)	8.9 (7.2–10)	10.3 (9–11)	8.8 (8.5–10.5)
Phosphorus, mg/dL	2.6	3.5	3.3
Creatinine, mg/dL	0.36	0.50	0.71
Alkaline phosphatase, U/L	402 (145–320)	626 (40–515)	234 (91–281)
iPTH, pg/mL (normal range)	52.9 (16–87)	13.5 (12–65)	58 (14–53)
25-OH-vitamin D, nmol/L	52	92	29
1,25 dihydroxy vitamin D, pmol/L (normal range)	40.9 (20–79)	45.6 (47.8–190.3)	49.9 (37–158)
**Urine metabolic markers**			
Calcium, mg/dL	1.9	1	0.7
Phosphate, mg/dL	14.7	46	258.5
Creatinine, mg/dL	9	9	89.2
TRP %	77.4	27	37.7
TMP/GFR mg/dL	2.00	0.94	1.25
**Burosumab therapy**			
Age at initiation	6 months	7 months	7 months
Dose at initiation, mg	10 (1.3 mg/kg)	10 (1.6 mg/kg)	10 (0.9 mg/kg)
Maximal dose, mg	30 (2 mg/kg)	30 (4.4 mg/kg)	30 (2.5 mg/kg)
Weight, kg %, z score	7.445 67 (0.44)	6.130 3 (−1.90)	10.800 100 (+2.80)
Length, cm %, z score	65.6 65 (0.40)	60.7 0 (−2.96)	69 82 (+0.90)
Weight-to-length %, z score	63 (0.34)	56 (0.14)	100 (+3.18)
**Serum metabolic markers**			
Calcium, mg/dL (range)	9.4 (7.4–11.7)	10.2 (8.5–10.5)	10.3 (8.5–10.5)
Phosphate, mg/dL	3.4	2.38	2.2
Creatinine, mg/dL	0.13	0.22	0.37
Alkaline phosphatase, U/L	536 (124–341)	647 (121.7–472.6)	474 (137–535)
PTH, pg/mL	36.6 (6.7–38.8)	27.3 (12–65)	83 (14–53)
25-OH-vitamin D, nmol/L	38	77	127
1,25 dihydroxy vitamin D, pmol/L (normal range)	63.4 (18–79)	45.6 (48–240)	135 (37–158)
**Urine metabolic markers**			
Calcium, mg/dL		1.81	2.8
Phosphate, mg/dL	31.3	4.5	41.8
Creatinine, mg/dL	8.7	4	59.3
TRP %	86.2	89.6	88
TMP/GFR mg/dL	2.93	2.13	6
Renal ultrasound	Normal at 5 yrs	Normal at 3 yrs 9 mo	Normal at 1 yr
Dental morbidity	Complete primary dentition with an open bite and deep grooves on molars	No complications	No complications

**Fig. 1:**
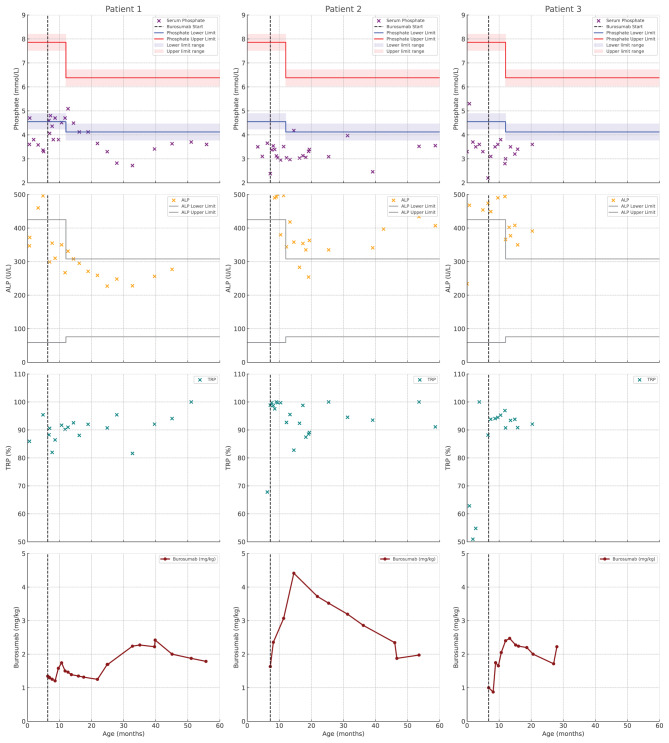
Evolution of biochemical response profile in 3 young patients with XLH treated with burosumab. Serum phosphate and alkaline phosphatase levels, tubular reabsorption of phosphate and burosumab dose (mg/kg). Age-specific reference ranges for phosphate and alkaline phosphatase are provided. The vertical dashed line indicates the start of burosumab treatment

Conventional treatment with phosphate supplements and calcitriol was initiated at 2 months of age. At 6 months, burosumab therapy was started at a dose of 10 mg (1.38 mg/kg), was administered at home by a nurse practitioner, and was titrated to a maximum of 40 mg (2.22 mg/kg). Burosumab treatment was initiated at our center following institutional approval, prior to national formulary listing for this age group. Despite stepwise escalation of burosumab dosing based on clinical and biochemical findings, serum phosphate levels remained below the lower limit of normal for age throughout follow-up. No adverse effects were observed, and the patient maintained consistent linear growth without evidence of orthopedic complications. The course of metabolic response to burosumab is shown in Fig. 1.

**Fig. 2:**
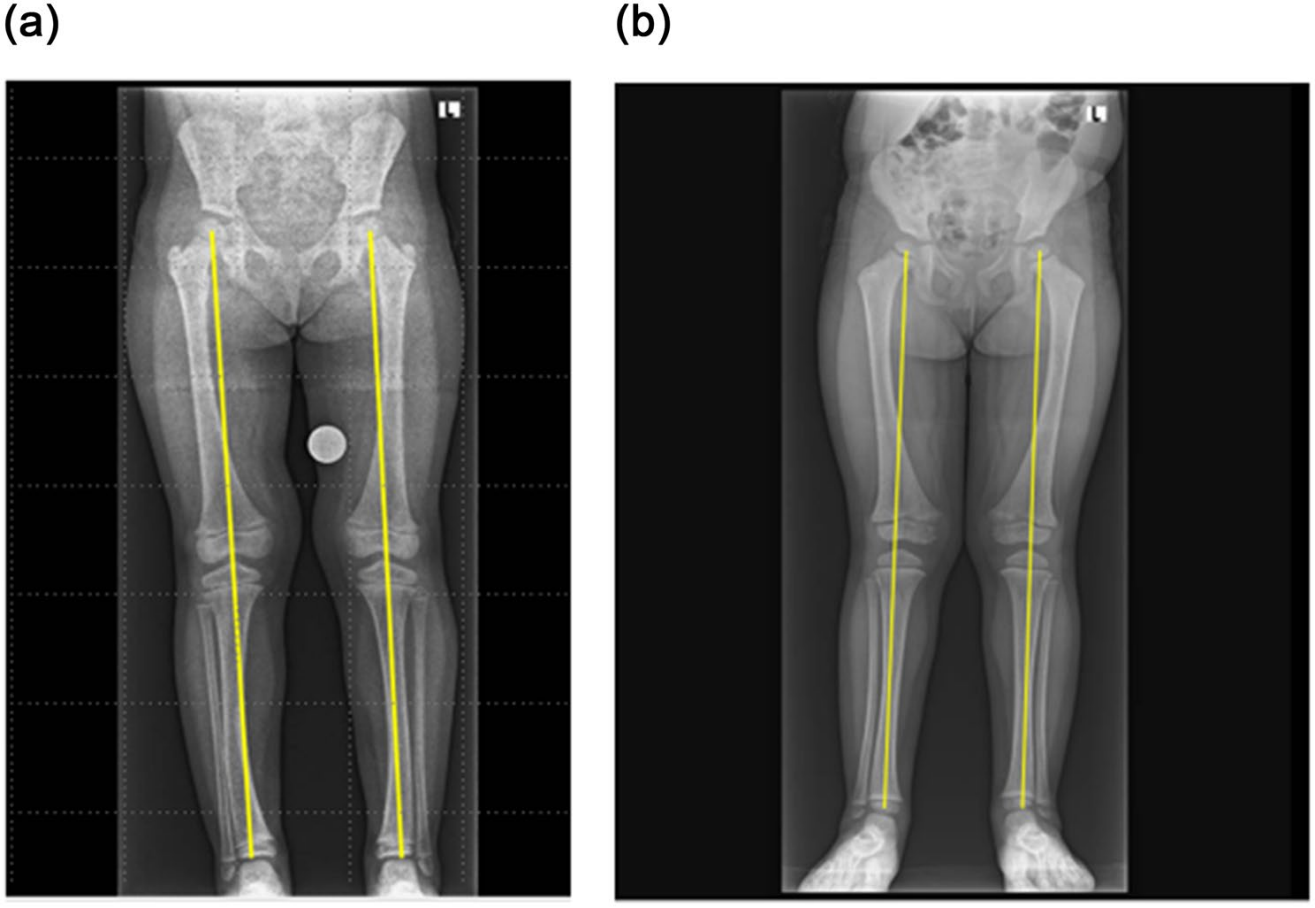
mechanical axis deviation (mad) in patients treated with burosumab. Panel **a**: patient 1, neutral mad after 4.6 years of burosumab treatment. Panel **b**: patient 2, neutral mad after 3 years of burosumab treatment. These radiographic images show the mechanical axis (highlighted by yellow lines) in two patients, demonstrating successful correction of lower limb alignment following burosumab treatment

At the age of 5 years, growth remained stable, with height consistently measuring between the 10^th^ and 25^th^ percentiles and weight at the 50^th^ percentile (BMI between the 85^th^ and 90^th^ percentiles). The patient’s mother reported significant improvement in bone pain and physical activity, although some residual musculoskeletal discomfort persisted. The skeletal outcomes showed a favorable RSS and neutral MAD (Table 3) without femoral bowing and with only mild tibial bowing (Fig. 2A). The neck-shaft angle was within normal limits (R146, L145; the normal range at the age of five years is 139 (±10) to 135 (±5) in early adulthood [17]. Evidence of enamel hypoplasia, delayed tooth eruption, and potential malocclusion persisted, which may be attributable to her underlying XLH or influenced by other factors, such as hygiene and nutritional status. There were no issues with compliance, and no known side effects were observed.

**Table 3:** Skeletal health and alignment: rickets severity score and mechanical axis deviation in XLH patients

Variable	Patient 1 5 yr 2 mo ♀	Patient 2 4 year 3 mo ♀	Patient 3 1 yr 10 mo ♀
Duration of burosumab treatment	4 yrs and 7 mo	3 yrs 3 mo	1 yr 3 mo
RSS at last assessment	0.5	1.0	1
Mechanical axis deviation	Rt 0, Lt 0, Zone neutral	Rt 0, Lt 0, Zone neutral	not relevant

**Patient 2.** This 4-year, 3-month-old female presented to the clinic at 3.5 months of age with hypophosphatemia. Her mother had been diagnosed with XLH at the age of 2.5 years and exhibited marked short stature with bowing of the legs, reaching an adult height of 143 cm (−3.12 SDS) despite conventional therapy. Biochemical evaluation of the infant revealed low serum phosphate and elevated alkaline phosphatase levels (Table 2). FGF23 was elevated at the time of diagnosis, supporting the clinical suspicion of XLH. Genetic testing was not pursued, as the patient’s mother declined it due to personal religious beliefs. This decision was respected in accordance with institutional ethical standards. Skeletal radiographic imaging confirmed the diagnosis of rickets. Conventional therapy was initiated at 3.5 months of age, and the patient was transitioned to burosumab therapy at seven months. Burosumab treatment began following institutional approval at our center, prior to its inclusion in the national formulary for this age group.

Although high-dose burosumab (2 to 4.4 mg/kg) was administered, her serum phosphate levels remained persistently below the age-specific reference range, ranging from 2.46 to 3.97 mg/dL. Alkaline phosphatase levels ranged from 254 to 363 U/L, with a single transient elevation to 4100 U/L, attributed to spontaneously resolving hyperphosphatasemia in infancy. The metabolic course and response to burosumab are presented in Fig. 1.

Growth deceleration became evident at 3.5 months of age, with length SDS of −1.08 and weight SDS of −1.47. At the time of burosumab initiation, body length and weight had declined to −2.56 SDS and −2.17 SDS, respectively. By 1.5 years of age, further growth faltering was observed, reaching nadirs of −3.38 SDS for length and −4.36 SDS for weight. Despite continued treatment, no substantial catch-up growth was noted. Marked improvements in both height and weight trajectories were noted only after the patient underwent adenoidectomy for obstructive sleep apnea at three years and nine months of age.

Skeletal features demonstrated a favorable outcome, as reflected by the RSS and MAD (Table 3, Fig. 2B), without femoral or tibial bowing. The neck-shaft angle was normal (R130, L132; normal range 140 ± 10 for age four years) [17]. No dental abscesses or abnormalities were reported.

**Patient 3** This 22-month-old female presented shortly after birth and was prenatally diagnosed with a *PHEX* mutation through whole exome sequencing conducted at the parents’ request, although there was no clinical indication. Postnatal blood tests revealed hypophosphatemia with normal alkaline phosphatase and calcium levels, providing biochemical evidence consistent with the diagnosis of XLH (Table 2). FGF23 levels were not measured prior to treatment initiation, as the patient was born at a center where this assay was not available.

Conventional therapy with oral phosphate supplements and active vitamin D analogs (calcitriol) was initiated following the diagnosis. At seven months of age, treatment was transitioned to burosumab, initiated at a dose of 10 mg (0.90 mg/kg) and titrated up to a maximum dose of 30 mg (2.50 mg/kg). The medication was administered at home by a nurse practitioner. After 15 months of burosumab treatment, serum phosphate levels remained below the age-specific reference range despite dose escalation. The metabolic course and response to burosumab are presented in Fig. 1.

At the age of 22 months, her growth was consistent, maintaining the 50^th^-75^th^ percentile for length and above the 97^th^ percentile for weight. Her knee RSS was +1 after 15 months of burosumab treatment (at 22 months of age) (Table 3). Despite genu varum that looked physiological, no femoral or tibial bowing was noted, and the neck-shaft angle was normal (R144, L144; the normal range is 141 ± 10 at three years of age) [17].

She achieved the motor milestone of independent walking at 14 months of age, which is within the expected developmental window. She did not experience any known side effects of the medication and demonstrated excellent adherence to the treatment regimen. No dental issues have been reported to date.

## Discussion

In this report, we describe three pediatric patients with XLH who began therapy during early infancy. Diagnosis through prenatal genetic testing or family history allowed timely intervention and enabled prompt initiation of treatment, likely contributing to improved growth and orthopedic outcomes.

Impaired growth is a hallmark of XLH, with untreated patients typically presenting with normal birth length followed by progressive growth deceleration in infancy and childhood [18, 19]. Two of the patients in our series (Patients 1 and 3) followed this expected pattern, maintaining linear growth in the lower percentiles, whereas Patient 2 experienced significant growth faltering despite early initiation of burosumab. The catch-up growth observed only after adenoidectomy for obstructive sleep apnea suggests that multifactorial contributors—beyond phosphate metabolism—may influence growth in patients with XLH. This underscores the importance of comprehensive evaluation and individualized care when assessing growth outcomes in this population.

Burosumab is currently approved for use in patients ≥6 months of age in Israel and the United States; however, limited data exist on its optimal use in younger infants, particularly those treated shortly after the age of 6 months. In our case series, all three patients were treated with burosumab doses that exceeded guideline-recommended starting doses (typically 0.8 mg/kg every two weeks). The rationale for these higher doses was twofold (1): persistent hypophosphatemia despite standard dosing, and (2) the clinical goal of achieving improvements in radiologic outcomes and linear growth during a critical developmental window. Specifically, in all three patients, serum phosphate levels remained below age-specific reference ranges even after several months of standard-dose therapy. This prompted stepwise dose escalation, consistent with the flexibility permitted in real-world management and supported by expert consensus that treatment should be guided by clinical, biochemical, and radiological response rather than serum phosphate normalization alone [20].

As understanding of XLH pathophysiology and burosumab response has evolved, therapeutic goals have shifted toward targeting improvements in functional outcomes—such as linear growth, radiographic healing, and physical activity—rather than strict normalization of phosphate levels. In our series, this approach was reflected in improved skeletal outcomes in all patients and stable or improved growth in two of the three, even in the context of persistently low serum phosphate levels. Importantly, decisions to exceed guideline doses were made under institutional oversight, in the context of careful safety monitoring and a multidisciplinary team approach.

Patients with XLH often experience bone deformities, such as lower extremity varus (bowed femurs and tibias, genu varum, and coxa vara), due to impaired phosphate metabolism and insufficient bone mineralization. These deformities can result in chronic pain, stunted growth, and mobility issues, which often require orthopedic interventions [1, 4–6]. In our case series, burosumab appeared effective in preventing lower extremity deformities. Although bone pain was not assessed by a structured questionnaire, the caregivers reported marked improvements in physical activity and reduced discomfort. Importantly, the two patients in whom the mechanical axis was assessable demonstrated neutral mechanical axis deviation, indicating improved lower limb alignment. This neutral axis supports the effectiveness of burosumab in enhancing skeletal health and preventing further deformities. None of the patients developed femoral or tibial bowing, and neck-shaft angles remained within normal limits for age, providing further support for the skeletal benefits of early treatment.

Dental health issues were absent in two of our patients, whereas one experienced dental complications despite early initiation of treatment with burosumab. This finding aligns with a previous report from our group that described a 5.5-year-old patient who sustained recurrent dental abscesses over three years of treatment with burosumab [21]. Brener et al. postulated that dental morbidity in XLH patients may be influenced by additional *PHEX*-related local mineralization inhibitors, such as osteopontin. This hypothesis is supported by the persistence of unique dental morphology, including unusually large pulp dimensions, in patients receiving burosumab therapy [21]. This variability may, however, reflect differences in individual genetic factors, adherence to oral hygiene practices, or compliance with supplement use.

All patients received their injected medication at home from a nurse practitioner, ensuring high adherence to treatment. There were no reports of any significant side effects from burosumab, suggesting that the drug is generally well tolerated in infancy. However, ongoing monitoring for potential side effects is essential, particularly given the long-term nature of the treatment.

The limitations of this case series include a small sample size, lack of a comparator group, individualized dosing, a relatively short follow-up period, and potential confounders in growth outcomes. Our series of three patients precludes our ability to provide any recommendations regarding dosage or underlying mechanisms, issues that warrant further investigation. Nonetheless, this study offers early real-world evidence on burosumab use in infancy and provides a foundation for future research. Strengths include detailed longitudinal data, early treatment initiation, and multidisciplinary care, which enabled close monitoring of outcomes and adverse effects.

## Conclusion

Our case series highlights the potential benefits of the initiation of burosumab treatment for XLH in early infancy by demonstrating improvements in growth, phosphate levels, and skeletal outcomes. While burosumab appears to be well tolerated, particularly in infancy, further research is needed to refine dosing strategies and assess the long-term safety and efficacy of burosumab in such young patients. Continued monitoring and individualized care are essential for optimizing outcomes and addressing the diverse needs of pediatric patients with XLH.

## Data Availability

The raw data supporting the conclusions of this article will be made available by the authors without undue reservation.
